# The Late M. Orfila

**Published:** 1853-09

**Authors:** 


### ECLECTIC DEPARTMENT,

### AND SPIRIT OF THE MEDICAL PERIODICAL PRESS.

*The late M. Orfila.*—In our last number, we mentioned the death of this distinguished philosopher, whose loss has been so universally lamented. The following brief biographical sketch of his illustrious career, which we find in the Medical Examiner, will be read with interest by every lover of science:

"The deceased was born at Mahon, in the island of Minorca, on the 24th of April, 1787, and could, as well expressed by M. de Salvandy, from the rocks of his country contemplate both empires, and one day choose between the two lands. He possessed within himself the genius peculiar to each soil—the fertile, bold and searching activity of the one, the firmness, patience, and enduring perseverance of the other. Orfila's mind was first engaged upon the study of mathematics, as he was intended for a maritime life, at a time when the French and Spanish fleets were co-operating in the East. He actually served a short time, but soon relinquished the sea, and from the pursuit of mathematics turned to the cultivation of the natural sciences. For this purpose he repaired to the University of Valencia, in the year 1804.

"Physics and chemistry were at that period at a very low ebb in Spain; and Orfila, not satisfied with hearing his professors stating that air and water were two elements, seized upon the works of Lavoisier, Berthollet, Fourcroy and others, and rose to the level of the discoveries of the day. But the University of Valencia was just then being threatened with abolition, on the plea of insufficient teaching; the professors provoked an inquiry, and put forward their pupils. Orfila greatly distinguished himself in the examinations, and so complete was his success, that the junta of Barcelona sent him to Paris at their own cost, to study chemistry, and the application of that science to the arts.

"He arrived in Paris in 1807, and soon took a great liking to a country which, for the subsequent forty years, became his adopted land. The war which soon broke out between France and Spain deprived him of his allowance from Barcelona, but he was temporarily supported by a member of his family. It should here be mentioned, that many years after this period, Orfila offered the junta his services, from gratitude at the patronage they had formerly granted him; but that body was in too disorganized a state to accept the honest man's disinterested proposals. Nor would his return to Spain have been a small sacrifice; for Vauquelin had welcomed him in his laboratory, and Fourcroy had entrusted to him the task of giving a few lectures on Organic Chemistry.

"Orfila now obtained the degree of Doctor of Medicine, and began to lecture privately on Chemistry, Forensic Medicine, and even Anatomy, (a kind of authorized grinding,) and in his first and small laboratory he laid the foundation of the new science of toxicology. We say *new*, and advisedly; for the books published before Orfila's time mention none of those delicate tests introduced by the great chemist; it is true, that even then certain poisons could be detected when dissolved in, or mixed with, water; but when these poisons were added to wine, milk, bile, &c., they were beyond the skill of the chemists of the day. Orfila's researches rendered investigations of this kind, and many others of equal importance, perfectly easy.

"Orfila was soon appointed corresponding member of the Institute, (a generic name given to the four academies taken collectively,) and in 1819, he was appointed Professor of Forensic Medicine at the Faculty of Medicine, especially by the influence of the celebrated Halle, who, though very ill, had himself carried to the medical school to vote for M. Orfila.

"It was at first supposed that lectures on Forensic Medicine at the Faculty would attract but few hearers; but Orfila's happy mode of teaching, and of captivating the attention of the pupils, rendered the subject peculiarly attractive. His language was lucid, without being too much studied; and he treated the most difficult questions with so much method and precision, that the mind readily followed him. He was especially fond of conveying knowledge by actual experiment, and gave his explanation with a clear and penetrating tone of voice, in well chosen periods, and without too much fire or any hesitation. He loved to teach and guide the pupils, and thus drew around himself numerous and attentive hearers during the four years he lectured on forensic medicine, and the twenty-nine which he devoted to medical chemistry. This success is the more flattering, as pupils are not *bound* to attend the lectures of any professor, and that the latter have a fixed salary, independent of the number of students who may attend them.

"The fame of Orfila was now daily spreading, and his works and teaching on toxicology, forensic medicine, and chemistry, testify to his immense labors; but he seemed to direct his whole energy to the detection of poisons. He looked upon the science of toxicology as composed of two principal divisions—one comprising the symptoms of poisoning, the consequent lesion of texture, and the medical treatment; the other, more exclusively chemical, embracing the detection of the poison either with a view of the discovery of an antidote, or for guiding the verdict of a jury. Both these branches were so highly cultivated by Orfila, that he became the oracle in the courts of law, and the dread of those who contemplated the treacherous administration of deadly substances.

"In 1820, the Academy of Medicine, as now constituted, was founded by Louis XVIII., and the original members were seventy in number. Orfila was one of them, and, strange to say, the youngest of all. He distinguished himself for the next thirty-three years as a fluent and able speaker, always defended the Academy in the contest which that learned body had to sustain, and, when chairman, acted with great tact and ability. Shortly before Orfila's death, only five of the original seventy members were left, and it was remarked that Orfila was again the youngest amongst them.

"The revolution of 1830, which carried Louis Philippe to the throne, became, for Orfila, the beginning of a series of prosperous days, in which wealth, honors, and the most envied appointments were showered upon him. But it may be said to his praise, that he rendered the greatest services, both to the state and to learning, in the several responsible and eminent posts which he successfully occupied.

"As Dean of the Faculty of Medicine, (in which dignity he succeeded the celebrated Antoine Duboise, father of the present accoucheur, Paul Duboise,) he used his best efforts, for a period of eighteen years, to introduce improvements of great importance in the medical school. Without neglecting toxicology or his professional duties, he began to root up abuses, and to make regulations which to this day call for the gratitude of the students. Lectures, by his endeavors, were now given with regularity; the pupils were subjected to a wholesome discipline; the miserable building opposite the Faculty, was demolished, and a new one, called 'Hopital des Cliniques,' erected in its place, where surgery and midwifery are carefully taught; new dissecting-rooms were substituted for the wretched places where anatomy was before cultivated; the Dupuytren museum was founded; a botanical garden was opened; and the anatomical and pathological collections of the faculty so enriched, that Orfila's name was attached to them as a lasting memorial of his wise and paternal government.

"But Orfila was now called upon to take an active share in the higher branches of the administration connected with learning and medical science; his influence became immense, but he contrived to make many friends, and to render the greatest services by his great aptitude for public business, his vast experience, quick glance, and mastery of details.

"Orfila successively took his seat in the Council General of Hospitals, and at the board of Public Instruction. The profession gained much by his elevation, for it was through his exertions that the preparatory schools of medicine in various large provincial towns were founded, and it was he who placed the examinations for the degree of Doctor of Medicine upon the present excellent footing.

"The subject of this memoir obtained the usual marks of high favor at the hands of the government, and was successively made knight, officer, and commander of the Legion of Honor. He became accustomed to guide and regulate, and when the exalted man was thrust into private life, by the destructive revolution of 1848, he felt the blow very acutely, and never recovered the shock.

"Among all the claims which M. Orfila has to the gratitude of his countrymen, there is one which will certainly be highly prized among us, and will remind our readers of those worthy members of our profession who, in England, have founded societies for the relief of the decayed medical man, his widow, or children. M. Orfila, at the period to which our narrative has arrived, founded a provident association for the benefit of medical men or their families; and for many years he was the president of the society. He constantly used his best efforts to promote its welfare, and succeeded in establishing it upon a sure and lasting basis. This society was not forgotten in the munificent legacies, which M. Orfila made shortly before his death, and to which we shall presently allude.

"But humane prosperity is fragile; M. Orfila's career of usefulness and benevolence was arrested by the fall of Louis Philippe; he lost all his appointments, (except his professorship, which could not be taken from him,) and it is said that these changes have, to the hour of his dissolution, preyed upon his mind, and even hastened his death.

"M. Orfila was not only displaced as dean of the faculty, but roughly called to account respecting the improvements and embellishments which had been made during his tenure of office; and the worthy and eminent man was, by envious detractors, sadly worried and taken to task. He endeavored to find comfort in scientific pursuits, and tried to regain his shattered health by a journey to the Pyrenees, but his friends saw that his powers were gradually failing.

"Only a few weeks ago, M. Orfila resolved to make all his benevolent legacies before his death, a sort of defiance (as justly mentioned by Professor Berard) which the leveler of all human greatness resented by speedily snatching at his prey. More than £4000 were thus given by M. Orfila, firstly to the Academy of Medicine, for prizes on medico-legal questions; secondly, to the Faculty of Medicine, for the preservation of the museum; thirdly, to the School of Pharmacy, for prizes; and to the Medical Benevolent Society for its praiseworthy purposes.

"A very short time after these munificent gifts were announced, and whilst grateful addresses were being sent from all parts of France, M. Orfila was attacked with pneumonia, soon after his last lecture, delivered on the 4th of March. Drs. Chomel, Andral, and Rostan, attended him with the greatest solicitude, but it was soon too plain that the illustrious patient must sink. He expired at half-past seven in the morning, on the 12th of March, and had, thirty-six hours previously, sought the comforts of the Christian religion.

"The funeral was as numerously attended as that of Dupuytren and Broussais; all the distinguished men in the arts, sciences, medicine, and the government, as well as crowds of students, accompanied the remains of this great and good man to their last and peaceful abode. The corners of the pall were held by M. Berard, Inspector of Public Instruction; M. P. Dubois, Dean of the Faculty of Medicine; M. Dubois, (d'Amiens,) Perpetual Secretary of the Academy of Medicine, and M. Bussy, Director of the School of Pharmacy. Orfila is buried close to the tombs of Lisfranc and Barruel, the latter of whom was his first and much revered teacher.

"The works left by M. Orfila, are the following: 1. On Forensic Medicine, with an appendix on Legal Exhumations, with twenty-six plates, four vols., fourth edition in 1848; 2. Elements of Medical Chemistry, two vols., eighth edition in 1851; 3. On Toxicology, two vols., fifth edition in 1852; and a great number of papers and reports on Toxicology, and Forensic Medicine, which have so largely contributed in placing both sciences on their present improved footing, and have gained for their author an imperishable fame."—*London Lancet.*

